# Cases of the Fever Which Prevails near Tho Drowned Lands, in Orange County, State of New-York, in a Letter from Peter H. Cole, House Physician to the New-York Hospital, to Dr. Mitchill, One of the Physicians of That Institution, Dated January 1st, 1810

**Published:** 1810-11

**Authors:** 


*Cases of the Fever which prevails near the Drowned Lands, in Orange County, state of New-York, in a letter from* Peter H. Cole, *House Physician to the New-York Hospital, to Dr.* Mitchill, *one of the Physicians of that Institution, dated January 1st,* 1810.

Sir,

As you requested, I transmit to you the cases of the patients labouring under fever from the Drowned Lands, near the Wallkill, which were received in the New-York Hospital, during the late term of your attendance. There is a peculiarity in them that, as you early observed, renders them worthy of a more distinguished place in medical history. The persons suffering under this disease have a yellowish or sallow complexion and eyes, and a certain haggard or ghastly look, very distinguishable from other maladies. These are so familiar to me, that I can, almost to a certainty, decide upon them when I meet them in the streets.

These low grounds, where the patients sickened, are in the immediate vicinity of the Wallkill. From their situation, lower than the outlet of the river, they have been subjected to inundations, and are almost always wet and marshy. Their extent amounts to many thousand acres. The bottom is a fertile mould, admirably adapted to the raising of hemp.

The draining of this valuable tract, called the "Drowned Lands," has been undertaken by a company under Legislative authority. Considerable progress has already been made in the work, which, though arduous, promises great advantage to private property, and a signal improvement of the public health.

The region, however, has been famous for its unhealthiness ever since the settlement of the country. During the hot season, copious exhalations fill the atmosphere, from solar evaporation; and then are precipitated in dense fogs, which gather during the latter part of the night, and overhang the earth until dissipated by the heat of the morning.

There is something very deleterious imparted to the atmosphere from this swampy surface, and it acts noxiously upon the human body during the time of sleep between midnight and sunrise, and during the hours of waking and fasting, until they get their first meal, or the cloudiness disappears. Indeed, few constitutions can long endure its malignity. Some of the most sturdy labourers are overpowered by it in a few days; and he has stouter nerves than commonly fall to the lot of man, to withstand it many weeks. The temptation of liberal pay draws to this unwholseme region a crowd of hardy labourers. As they sicken and become disabled, a new set is ready, under the same inducement, to take their places. These stand as long as their strength lasts, and in their turn, are succeeded by fresh hands. And by these means, the important and desirable work of draining the Drowned Lands, is carried on.

It is proper to observe, that in addition to the high wages, are included board and lodging, with a daily allowance of about a pint of whiskey. This appears to have been entirely consumed, and many of them have been known to buy an additional quantity from the retailers.

The disease which I have described cannot fail to afford instructing analogies with fevers which arise in the sea-ports. Indeed, the conclusion seems irresistable, that febrile diseases, whether arising in the interior situations, or on the sea coast, are of kindred natures; and run into each other by shades and gradations, better known to the experienced observer in practice, than capable of being reduced to the rules of nosology.

In the cases I have recorded, the facts were derived from the mouths of the persons respectively, while they were under your prescription. The general debility, the anomalous form of fever varying from the continued to the quotidian and tertian, the indications of disordered liver and spleen, the yellow hue of the skin and eyes, the occasional dropsical effusions, together with the obstinacy and violence with which the disease harassed those whom it invaded, are matters of prominent regard in its history. If the account I give you shall afford any thing new in medical research, or shall have delineated in a way that is in the smallest degree instructive, the endemic distemper of that noted district, I shall think the pains I have taken are well rewarded.

### Case I.

William Holmes, Englishman, aged 37—has been four years in the United States, chiefly in Boston, as a hardware dealer. Went to the outlet to work in June, and continued to labour for six weeks, at twelve dollars a month. He laboured with a crowbar and spade, and in carrying stones. Was then seized with anorexia, nausea, headach, and pains in the back and limbs. After a week's lingering, with loss of appetite, and increasing debility, he came to New-York, by the way of Goshen and Newburgh. After a week's continuance in Batavia-lane, he was received into the Hospital on on the 11th August. At that time he had become so much debilitated that he could scarcely walk. His ankles were œdematous, and there was a troublesome pain in the left hypochondrium. His febrile symptoms were exceedingly anomalous, and the paroxysms, which had never been preceded by chilliness while he was in the country, assumed, sometimes, the type of a coldish stage, which terminated in a hot and sweating stage, of irregular returns, after his reception into the Hospital.

He took a mercurial cathartic, was blistered over the left hypochondrium, and ordered columbo and ginger, decoct. cort. peruv. and acid sulph.; under which remedies he has gathered considerable strength. His recovery proceeded gradually, under the above remedies and occasional emetics, until the 15th October, when his mouth became affected by the use of mercurial purgatives, which removed the occasional chills, swelled feet, and pain in the left hypochondrium. He was discharged, cured, Dec. 4th.

### Case II.

Patrick M'Gennigal, Irishman, aged 30—has been about four years in the United States. Was at the drowned lands last year, and was taken sick after nine weeks labour, at twelve dollars a month. The form of the disease was that of a quotidian and tertian intermittent, commencing with a cold and shivering fit. Notwithstanding which, he continued labouring long enough to make up the whole of three months work, by returning to service as soon as a cessation of the disease, from time to time, would permit. After this job was over, he was troubled with the paroxysms, by intervals, during the whole winter; sometimes escaping it for a week or more, and seized with it again. Notwithstanding all this suffering, he engaged in the employ, once more, this season, for the lure of fourteen dollars a month. He began to labour about the beginning of August: his employ was ditching, digging, and carrying earth and stone. While he was engaged in this business, he was frequently immerged in mud as high as the knees; and sometimes, when making the dam, he was in as deep as the waist. This year, he was taken sick about the 10th August. The first symptoms were a swelling of the legs and general debility, afterwards a chilliness and shivering, followed by severe hot fits. These recurred every day, for about fourteen days, when he removed to New-Windsor. There he languished for three weeks; the disease assuming the tertian type, and finding himself rather better, got on board a sloop and came to New-

 York,

York. From the sloop he came straight to the Hospital. Was admitted on the 8th Sept.

His situation was still febrile, with daily returns of his cold and hot fits. His legs, thighs, and body were swelled, with anasarcous effusion. There was heaviness and pain in the right hypochondrium; an uneasy sensation in the shoulder, and under the scapula of the same side.

For this patient, cream of tartar, in small doses, was for some time pursued, with little benefit. On the 20th he was put upon the use of the following decoction, rad. seneka ʒij. bac. juniper ℥i. fol. digital gr. x. aquæ fervent. ℔j. Simmer them for a few minutes, and after being strained, he took two table spoonfuls every hour, increasing the dose according to circumstances. Under this, absorption took place, and much evacuation by urine was the consequence.

On the 24th he took a pill composed of calomel gr. 1, pulv. scillæ gr. ij. three times a day. Under this, a further discharge of the accumulated water took place, and the hepatic disease was considerably relieved. Under the operation of this remedy, his mouth, in seven days, grew sore.—Since he was put upon the decoction and pills, he has had no return of the febrile fits. The salivation began about the 1st October, and during its continuance, he gradually and sensibly mended. On the 14th, a blister was applied to the affected side, which has removed the greater part of the remaining pain. He was discharged, cured, on the 23d October.

CASE III.

Martin Joyce, Irishman, aged 33—has been eleven years in the United States. Chiefly engaged as a seaman, in Portsmouth and Boston. Arrived at the Drowned Lands 15th August, induced by the wages, of being found and paid fifteen dollars a month. Was engaged in hauling rocks beside the ditch or drain. Was taken sick on the 11th day after he began to labour. His symptoms were headach, pain in the back and limbs, and continued fever, which lasted seven days, when he was seized with a chill which was severe, and was succeeded by a hot and sweating stage. Since that time he had a daily return of the fit in the form of a regular quotidian until the 10th October.

After lingering about three weeks near the Drowned Lands, he came to New-York, by the way of Goshen and Newburgh. On the 3d October he was received into the Hospital.

His paroxysms had regularly invaded him while he travelled. After his first seizure he had taken an emetic, a cathartic, cathartic, cort. peruv. and wine, besides a variety of popular remedies.

When received into the Hospital, he complained chiefly of the severity of his paroxysms, and of the debility caused by them. The remedies employed were decoct. cort. peruv. and serpentar. arsenical solution, and anodyne draughts to prevent the severity of the paroxysms.

His paroxysms continued to visit him, in the quotidian type, until the 14th October; they then left him until the 24th, when they returned with great irregularity until the 1st November, when it left him. He was discharged cured on the 18th November.

### Case IV.

Thomas Burns, Irishman, aged 30—has been in the United States about four years, chiefly employed as a seaman, from the port of Boston. Fearing impressment from the British, if he went to sea, he concluded to go to the Drowned Lands; and arrived there in the beginning of September. He laboured there sixteen days, for fifteen dollars a month, at digging and wheeling earth, but not exposed to the water, above the ankles. The water was warmish and not cold like spring water. He left this place and went to Wardsbridge, and was taken sick at the end of the first half day. The first symptoms were headach, want of appetite, pain in the back of the neck, and chilliness; then fever and sweating, as usual, succeeded. The paroxysms returned every day. After five days recurrence of fits, came in a waggon to Newburgh, whence he came to New-York in a sloop. He arrived on the 2d October, and was received into the Hospital the same day.

At that time, he was visited every day with a chill and fever; rheumatic pains in the back and shoulders, and complained of soreness, and aching in the left hypochondrium, about the region of the spleen.

He took an emetic, cathartic, and several of Dover's powder opiates were administered to check the onset of the paroxysms; and cort. peruv. given at the intervals.

His paroxysms returned at irregular periods, sometimes every other day, and at other times every sixth or seventh day. He was discharged cured on the 29th November.

### Case V.

Thomas Boyd, New-Yorker, aged 36, labourer; went to work at the Drowned Lands on the 19th August, and laboured laboured there on the dry soil, with the barrow and drag. At the end of the fourth week after his arrival, he was seized with pain in the head, giddiness, want of appetite, pain in the left hypochondrium, and fever. After three days he experienced a chill. This returned every other day, and was succeeded by regular hot and sweating stages.

He then left the place and returned to New-York, by the way of Goshen and Newburgh. He arrived here on the 1st October, and was received into the Hospital on the 7th, having staid at a house near the Fly-market in the intervening time, and suffered paroxysms each alternate day.

On being admitted, he complained of loss of appetite, headach, general debility, and tertian paroxysms. On the 10th the paroxysms became quotidian, and continued to recur daily until the 14th, but though they came on more frequently, they were less violent.

The remedies were an emetic, diaphoretic powders, arsenical solution. decoc. peruv. with serpent. and acid sulph.

His fever continued in the form of a quotidian and sometimes tertian type, until the 18th November. His appetite and strength improved rapidly. He was discharged cured on the 4th December.

### Case VI.

James Derrick, Irishman, aged 29—has been eight years in America, chiefly in New-York, in the occupation of a carpenter. He arrived at the outlet, five miles from Goshen, the 8th September, and was to receive fourteen dollars a month. He was employed at the barrow and drag; and was taken sick on the 15th. He had no chill, but anorexia, pains in the head and limbs, and a kind of feverishness, which for several days permitted him to do partial labour. Finally, however, he was obliged to give it up. On the 5th October, he came off, and proceeded, on foot, by way of Goshen, to Newburgh, from whence he arrived in a sloop at New-York on the 7th, and was received into the Hospital on the 10th.

He took an emetic and a peppermint draught before he left the outlet.

He complained, on being received, of pain in the breast, and of general exhaustion. On the 14th he, for the first time, experienced somewhat of chilliness. Under the application of a blister to the breast, a mercurial cathartic, diaphoretic powders and decoct. cort. peruv. and serpent. he is doing well.

His fever, during the present month, assumed in a considerable degree, the continued form; at intervals quite delirious, lirious, and in action resembled an idiot. About the beginning of November dropsical swellings appeared in the legs and feet, which were shortly removed by the free use of crem. tart. digitalis and squills. Cort. peruv. used freely to restore strength. Was discharged cured Nov. 19th.

### Case VII.

Patrick Hanling, Irishman, aged 43—has resided in New-York about 22 years. Went to work at the Drowned Lands about the 10th September, in company with six others, to take the benefit of fourteen dollars a month as wages. Was employed as a digger and ditcher, and much of the time was in the water up to the knees. On the 10th day of his labour was seized, while at work, with severe headach, pain in the back and limbs, and was obliged to leave off and go to bed. Then came on a violent chill, which made him wish for all the blankets in the house to cover him. This was followed by a hot and sweating stage. The fits returned every day for nine days. He then came to Goshen, thence to New-Windsor, and from thence to New-York, where, after continuing about a week in lodgings in Ferry-street, he came to the Hospital. In the latter place he took an emetic, cathartic, and some doses of cort. peruv. by which he was so far relieved that his paroxysms left him.

He was admitted October 13th, complaining of universal debility and griping pains. His pains abated in a little while without any remedy. He was put upon the use of cort. peruv. for his debility. He was discharged cured the 28th October.

### Case VIII.

John Riley, Irishman, aged 40—has been nine years in America, chiefly in New-York, in the capacity of a labourer. He arrived at the outlet about the 9th August, with three in company, and was to have fourteen dollars a month. He was employed at the barrow, dry digging, and crowbar. He was seized about the 9th September, with pain in the head and extremities, which was followed with a severe chill, that commenced about noon, and was succeeded by a hot and sweating stage. He continued working by the half day, notwithstanding his return of fever every day. A few days before he left the outlet his fever put on a tertian type. His appetite was vigorous at the beginning of his sickness, but was losing as the disease advanced. After working about two months at the outlet, he came to New-York by the way of Goshen and Newburgh. He took an emetic while there.

He

He was received into the Hospital on the 17th October with a tertian intermittent; pain from the left clavicle, extending down to the region of the stomach; cough, some difficulty of breathing, and great debility. He attributes the pain and cough to exposure to cold while on board of the sloop on his passage down the river. On receiving him he was bled, blistered, and took powders composed of glauber salts and ant. tart. which soon removed his pain, cough, and difficulty of breathing. He used the decoct. cort. and serpent. His strength improving very fast, he was discharged cured on the 24th October.

### Case IX.

James Cole, Irishman, aged 27, seaman; has been in America about seven years; sailing partly from New-York, and partly from Portsmouth. Arrived at the Drowned Lands about six weeks ago, and worked about five miles from Goshen. His occupation was brickmaking. In a fortnight after he got there he was taken ill; was seized with a chill while at labour. This was followed by a violent fever, and profuse sweating. The fever lasted about three weeks without any recurrence of the chill, and was of the continued kind; then the shivering returned, and he has suffered a daily paroxysm, regularly from that time to the present.

While there he took an emetic and a cathartic; and no other medicine, either then or since. Finding himself exhausted too much to work, and being destitute of money, he got in a waggon and came to New York, through Goshen and Newburgh. He got to town in a sloop on the afternoon of the 22d, having had his fits every day during the voyage. He had no symptoms of dropsical and hepatic affection; but the bilious and woeful countenance is strongly marked in him, and in addition to his weakness and quotidian attacks, complains of pains and increasing heaviness in the left hypochondrium, which obliges him to lie on the right side.

He was blistered over the left hypochondrium. He also used the Peruvian bark in different forms, and occasionally emetics, with very little relief. He was then ordered the calomel pill, which affected his mouth, and caused the fever to disappear. He was discharged cured on the 16th December.

